# Supplementary material for: EnzML: multi-label prediction of enzyme classes using InterPro signatures
Source: BMC Bioinformatics. 2012 Apr 25;13:61. doi: 10.1186/1471-2105-13-61 (PMC3483700; doi:10.1186/1471-2105-13-61)
Supplement: Addtional file 5 — The Java code to format the data files, evaluate and predict. The file enzml_java_code.tar.gz contains the Java code used to format database data to ARFF and XML formats, to execute cross and train-test (jackknife) evaluations and to record evaluation results to database. More information is included in the readme.txt file and the Javadoc files. The code can be used with a MySQL database. To use a different database software, other JDBC drivers might be required. [file 1471-2105-13-61-S5.gz › java_code/enzml2011/doc/uk/ac/ed/inf/enzml/Initialised.html]

Initialised


---


|  |  |  |
| --- | --- | --- |
| **Overview** \| **Package** \| **Class** \| **Use** \| **Tree** \| **Deprecated** \| **Index** \| **Help** | |  |
| **PREV CLASS**   **NEXT CLASS** | **FRAMES**    **NO FRAMES**     **All Classes** |  |
| SUMMARY: NESTED \| FIELD \| CONSTR \| METHOD | DETAIL: FIELD \| CONSTR \| METHOD |  |


---


## uk.ac.ed.inf.enzml Class Initialised

```
java.lang.Object
  uk.ac.ed.inf.enzml.Initialised
```

**All Implemented Interfaces:**: Initialisable

**Direct Known Subclasses:**: Arff, ArffProperties, AttributesFilter, MulanArffRecord, MulanCrossExperimenter, MulanPredict

---

``` public class Initialised extends java.lang.Object implements Initialisable ```

---

| **Field Summary** | |
| --- | --- |
| `boolean` | `m_initialisationIsCorrect` |


**Constructor Summary**

`Initialised()`


| **Method Summary** | |
| --- | --- |
| `boolean` | `initialisationIsCorrect()` |
| `void` | `setInitialisationIsCorrect(boolean isCorrect)` |

**Methods inherited from class java.lang.Object**

`equals, getClass, hashCode, notify, notifyAll, toString, wait, wait, wait`

**Field Detail**

### m\_initialisationIsCorrect

```
public boolean m_initialisationIsCorrect
```


**Constructor Detail**

### Initialised

```
public Initialised()
```


**Method Detail**

### initialisationIsCorrect

```
public boolean initialisationIsCorrect()
```

:   **Specified by:**: `initialisationIsCorrect` in interface `Initialisable`

---


### setInitialisationIsCorrect

```
public void setInitialisationIsCorrect(boolean isCorrect)
```

:   **Specified by:**: `setInitialisationIsCorrect` in interface `Initialisable`


---


|  |  |  |
| --- | --- | --- |
| **Overview** \| **Package** \| **Class** \| **Use** \| **Tree** \| **Deprecated** \| **Index** \| **Help** | |  |
| **PREV CLASS**   **NEXT CLASS** | **FRAMES**    **NO FRAMES**     **All Classes** |  |
| SUMMARY: NESTED \| FIELD \| CONSTR \| METHOD | DETAIL: FIELD \| CONSTR \| METHOD |  |


---
